# Effects of a physically active gamification program on cognitive function in primary school children

**DOI:** 10.3389/fspor.2026.1967341

**Published:** 2026-10-08

**Authors:** María del Carmen Carcelén-Fraile, Amanda Camacho-Illana, Alba Rusillo-Magdaleno, Alberto Ruiz-Ariza

**Affiliations:** 1 Department of Educational Sciences, Faculty of Educational Sciences, University of Atlántico Medio, Las Palmas de Gran Canaria, Spain; 2 Department of Didactics of Musical, Plastic and Corporal Expression, University of Jaén, Jaén, Spain; 3 Institute for Transfer and Research (ITEI), International University of La Rioja (UNIR), Logroño, Spain

**Keywords:** active gamification, attention, cognitive function, memory, physical activity, primary school children

## Abstract

**Background:**

Regular physical activity is associated with improvements in cognitive function during childhood. Integrating physically active learning strategies, such as active gamification, into school settings may enhance cognitive processes while increasing student engagement. However, evidence from randomized controlled trials in primary education remains limited.

**Aim:**

This study aimed to examine the effects of a physically active gamification program on concentration, selective attention, memory, and creativity in primary school children.

**Methods:**

A cluster-randomized controlled trial was conducted with 120 children aged 8–11 years from six intact classes. Classes were randomly assigned to an experimental group, which completed a 12-week physically active gamification program during physical education lessons, or to a control group, which followed the standard curriculum. Concentration and selective attention were assessed using the D2 Test, creativity with the CREA Test, and immediate visual memory using a task adapted from the Reynolds Intellectual Assessment Scales (RIAS). Intervention effects were analyzed using linear mixed-effects models, with group, time, and the group × time interaction as fixed effects and classroom included as a random effect to account for clustering. Effect sizes were calculated using Cohen's *d*.

**Results:**

Significant group × time interactions were observed for concentration, selective attention, immediate visual memory, and creativity, indicating greater improvements in the experimental group than in the control group. Estimated marginal means showed that concentration increased from 58.43 to 63.68 in the experimental group, while remaining relatively stable in the control group (60.08 to 60.25). Similar intervention-related improvements were observed for selective attention and immediate visual memory, with the latter increasing from 9.37 to 11.62 in the experimental group compared with 9.47 to 10.20 in the control group. Creativity increased markedly in the experimental group (44.22 to 53.00), whereas it remained relatively stable in the control group (45.47 to 44.69). All group × time interactions were statistically significant (*p* < .05).

**Conclusions:**

A 12-week physically active gamification intervention improved several cognitive functions in primary school children, including attention, memory, and creativity. These findings support the integration of physically active gamification approaches into school-based physical education to promote cognitive development.

## 1 Introduction

Cognitive development during primary education, particularly between 8 and 11 years of age, is a critical period for acquiring skills that underpin academic and social functioning (1). Key cognitive processes, including concentration, selective attention, creativity, and memory, are essential for learning, problem solving, and adapting to educational demands. Concentration enables students to maintain focus on tasks, whereas selective attention allows them to distinguish relevant from irrelevant information, a particularly important ability in increasingly distraction-rich learning environments (2, 3).

Furthermore, creativity, understood as the ability to generate novel, original and useful ideas, is a key competence in the 21st century, not only to solve problems in an innovative way, but also to foster critical thinking and cognitive flexibility (4–6). Meanwhile, memory, in both its short- and long-term dimensions, is essential to store, process and retrieve information, allowing students to effectively connect prior knowledge with new learning (7, 8). The interaction between these cognitive abilities forms the basis for meaningful and sustainable learning throughout life.

However, increasing technological distractions and external stimuli make the development of these skills more challenging (9). As traditional teaching approaches may be insufficient to sustain students' attention and engagement, innovative pedagogical strategies are needed to promote motivation and cognitive development (10, 11). Recent frameworks propose that physically active learning influences educational outcomes through both general executive functions and domain-specific cognitive processes. Although gains in general cognition do not always translate into subject-specific learning, classroom-based evidence suggests that physically active interventions can also improve domain-specific skills, such as mathematics-related inhibitory control (12, 13).

In this sense, active gamification is presented as a promising tool (14). Gamification, defined as the incorporation of game elements in non-playful contexts, seeks to transform educational activities into dynamic, attractive and participatory experiences (15). By integrating game dynamics in the classroom, such as challenges, rewards, narratives and roles, an environment is created that stimulates students' intrinsic motivation, favoring more active and meaningful learning (16, 17). In addition, active gamification, which emphasizes movement and physical interaction, adds a kinesthetic component that can contribute not only to cognitive development, but also emotional and social development (18).

From a theoretical perspective, these effects can also be interpreted through the lens of embodied cognition and cognitive load theory. Embodied cognition suggests that cognitive processes are deeply rooted in bodily interactions with the environment, meaning that movement-based activities can enhance learning by integrating physical and cognitive experiences (19). At the same time, cognitive load theory highlights that instructional designs must carefully balance task demands, as excessive physical or cognitive demands may overload students' processing capacity and hinder learning. Therefore, physically active and gamified interventions should be designed to optimize cognitive engagement without exceeding learners' cognitive load.

Previous studies have suggested that active gamification has the potential to improve skills such as concentration, by keeping students focused on specific tasks through clear objectives and immediate rewards (20). It can also strengthen selective attention by requiring children to discriminate between relevant and irrelevant stimuli in a gaming context. Furthermore, game-like dynamics often foster creativity by allowing students to explore original solutions and collaborate in problem solving (21). Finally, the repetition and association of gamified activities can have a positive impact on memory, helping to consolidate learning more effectively (22).

The present study examined the effects of an active gamification intervention on concentration, selective attention, creativity, and memory in primary school students. A 12-week school-based program was implemented to evaluate the influence of gamified activities on these core cognitive processes. Although these processes underpin learning, they do not directly measure academic or subject-specific achievement. Accordingly, the study focused on general cognitive outcomes rather than academic performance, contributing to the evidence on active gamification as a pedagogical approach to support cognitive development in children aged 8–11 years.

## 2 Methods

### 2.1 Study design

This study was conducted to analyze the effects of a gamification program on cognitive development in primary school children. This research was approved by the Ethics Committee of the Universidad del Atlántico Medio and its design complies with Spanish legislation on clinical research in human beings (Law 14/2007, of July 3, on Biomedical Research), the regulations on the protection of personal data (Organic Law 3/2018) and the principles established in the Declaration of Helsinki (version 2013, Brazil).

### 2.2 Participants

Of the 129 students initially recruited, 120 met the eligibility criteria and were included in the study. Participants were enrolled in six classes across three grades of Primary Education (3rd, 4th, and 5th grades) at one primary school in Spain. The 120 eligible participants were distributed as follows: class 3A (*n* = 20), class 3B (*n* = 19), class 4A (*n* = 20), class 4B (*n* = 20), class 5A (*n* = 21), and class 5B (*n* = 20). Classes 3A, 4B, and 5B were allocated to the experimental group (EG; *n* = 60), whereas classes 3B, 4A, and 5A were allocated to the control group (CG; *n* = 60).

The inclusion criteria were: (i) age between 8 and 11 years; (ii) no medical contraindication to physical activity; (iii) no intellectual disability that could prevent understanding or compliance with the study procedures; and (iv) written informed consent from parents or legal guardians (Figure 1). Students who did not meet these eligibility criteria were able to attend the intervention with their classmates but were not included in the analysis.

**Figure 1:**
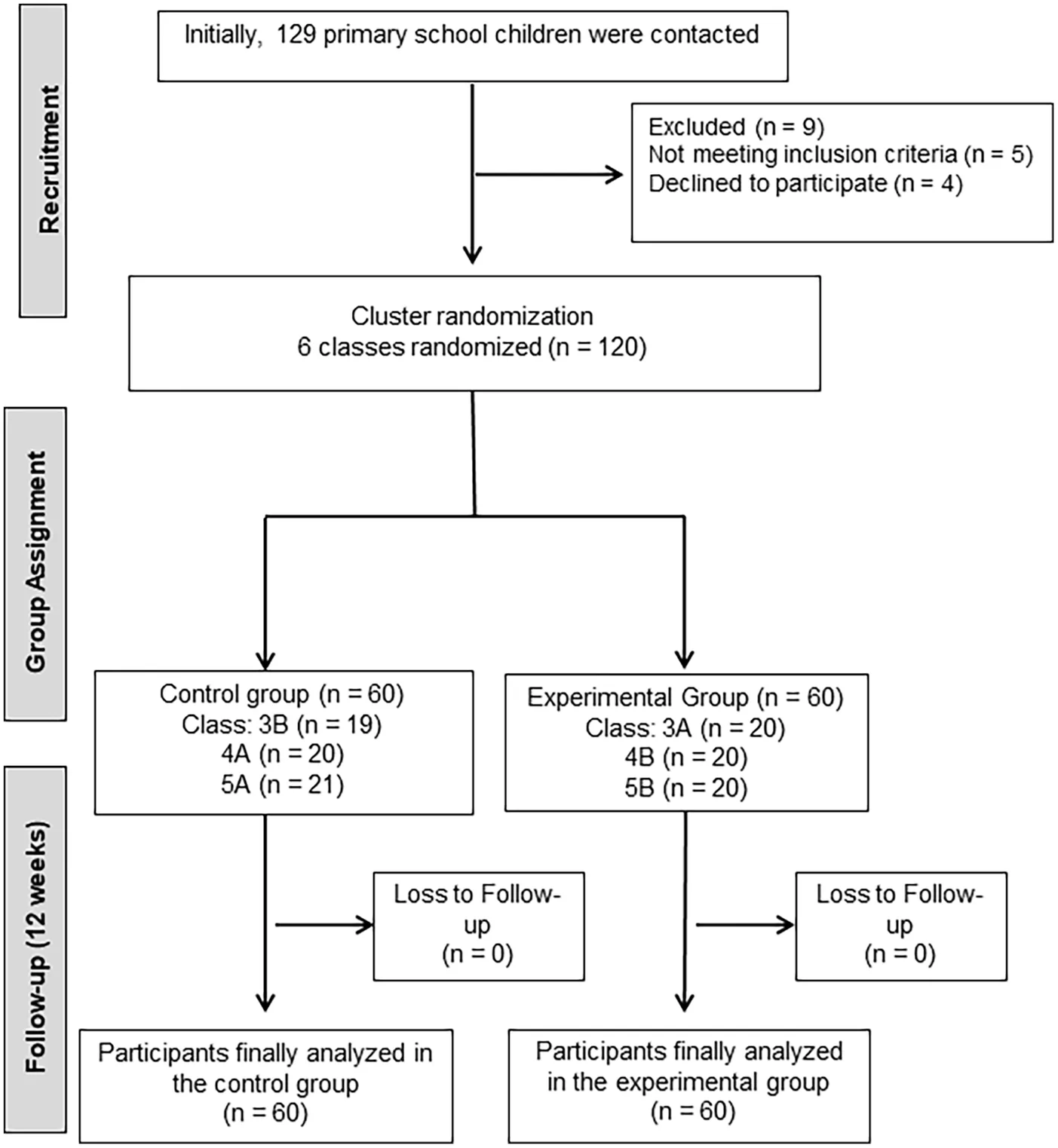
CONSORT flow diagram of cluster and participant progression through the study.

### 2.3 Measures

#### 2.3.1 Concentration and selective attention

Concentration and selective attention were assessed using the Spanish version of the D2 Test (23). The test was administered individually in the participants' regular classroom by a member of the research team. Before testing, the examiner explained the task and provided examples to ensure that each participant understood the instructions. Participants were then instructed to scan each row and identify all target letters (“d” with two marks) while ignoring distractors (“d” and “p” with other mark combinations). Participants were given 20 s to complete each row, after which they proceeded to the next row following the examiner's instruction. Concentration was assessed using the Concentration Index (CON), calculated as the total number of correctly identified target items (TA) minus commission errors (C) (CON = TA − C). Selective attention was assessed using the Total Effectiveness score (TOT), calculated as the total number of items processed (TR) minus omission (O) and commission (C) errors [TOT = TR − (O + C)]. Higher CON and TOT scores indicate better performance. The D2 has demonstrated excellent reliability (Cronbach's *α* and test–retest coefficients = 0.90–0.97) and adequate convergent and divergent validity (24). In the present sample, internal consistency was acceptable (Cronbach's *α* = 0.803).

#### 2.3.2 Creativity

Creativity was assessed using the Creative Intelligence Test (CREA), a standardized instrument designed to assess creative potential through the generation of questions in response to a visual stimulus. Participants were presented with stimulus Plate C and instructed to generate as many questions as possible about the image within a fixed time limit of 4 min. The CREA is based on the premise that the ability to generate multiple questions in response to an ambiguous visual stimulus reflects cognitive flexibility, curiosity, and divergent thinking associated with creative potential. Responses are scored according to the standardized CREA scoring criteria, yielding a quantitative creativity score, with higher scores indicating greater creative potential. The CREA has demonstrated adequate reliability (Cronbach's *α* = 0.875) and validity (25). In the present study, internal consistency was acceptable (Cronbach's *α* = 0.771).

#### 2.3.3 Immediate visual memory

Memory was assessed using a 1 min *ad hoc* test adapted from the Spanish version of the Reynolds Intellectual Assessment Scales (RIAS) (26). Participants viewed a poster displaying 15 randomly selected Spanish playing cards for 20 s and then had 40 s to recall as many cards as possible on a standardized response sheet (score range: 0–15). Two different sets of 15 cards were used at pre- and post-test to minimize potential recall and practice effects resulting from repeated exposure to the same stimuli. The administration procedure was identical at both assessment points. Before testing, all participants demonstrated familiarity with the Spanish deck. This procedure has been used in previous research, showing excellent test–retest reliability (48 h; *r* = .919) (27). Internal consistency in the present sample was acceptable (Cronbach's *α* = .802). Given the immediate-recall nature of the task, the resulting score was interpreted specifically as an indicator of immediate visual memory rather than as a comprehensive measure of the broader construct of memory.

### 2.4 Intervention

The EG participated in a 12-week physically active gamification program consisting of three 45 min sessions per week, delivered during regularly scheduled Physical Education lessons. Each session comprised a 10 min warm-up, a 30 min main activity, and a 5 min period devoted to rewards and ranking updates. The intervention was informed by Gagné's Instructional Design Model (28) and Keller's ARCS Model (29), integrating game mechanics with physical activity to promote attention, motivation, active participation, and learning retention.

The program was organized into three progressive four-week thematic phases: Explorers in Action (weeks 1–4), Movement Heroes (weeks 5–8), and Guardians of Health (weeks 9–12). Across these phases, students participated in progressively challenging activities involving coordination, balance, agility, speed, endurance, throwing, and other fundamental motor skills. These physical tasks were embedded within cooperative and problem-solving challenges involving spatial orientation, route planning, decision-making, observation, strategy, riddles, and collaborative goal achievement.

Gamification was integrated throughout the intervention using thematic narratives, team-based missions and challenges, points, rewards, progression, rankings, and achievement recognition. Rather than being implemented as separate cognitive-training activities, the cognitive demands were embedded within the physical challenges and constituted part of the gamified experience. A detailed description of the sessions, including physical tasks and motor objectives, cognitive processes addressed, and the specific gamification dynamics and mechanics employed, is provided in Supplementary Table S1. The control group (CG) followed the standard Primary Education Physical Education curriculum, including fundamental motor skills, team sports, coordination, and general fitness activities delivered by the same teachers. Session frequency and duration matched the experimental group (45 min, three times per week for 12 weeks), ensuring equivalent physical activity exposure. Unlike the experimental group, the CG received no gamification elements (e.g., narratives, points, rewards, or progressive challenges), making the pedagogical approach the primary difference between groups. Importantly, the problem-solving, route-planning, riddle-based, strategic, and collaborative tasks included in the experimental program were implemented entirely within the gamified framework rather than as separate cognitive activities. These tasks constituted the missions and challenges through which the narrative, progression, team dynamics, points, rewards, and other game elements were operationalized. Thus, the experimental condition represented an integrated active gamification approach rather than the addition of independent cognitive-training activities to Physical Education.

### 2.5 Procedure

Participants were recruited from a public primary school located in eastern Andalusia, Spain. The school was situated in a predominantly middle-socioeconomic context and followed the standard school schedule from 9:00 a.m. to 2:00 p.m. All study procedures were conducted within the regular school timetable, and the intervention was implemented during the Physical Education lessons routinely scheduled for each participating class. Following written informed consent from parents or legal guardians and eligibility assessment, the six participating classes were randomized to either the experimental group (EG) or the control group (CG) using sealed, opaque envelopes prepared by an independent researcher, ensuring allocation concealment. Randomization was performed at the class level, with three classes allocated to each study condition. Classes 3A, 4B, and 5B were assigned to the EG, whereas classes 3B, 4A, and 5A were assigned to the CG. As randomization was conducted at the class level, students remained in their usual class groups throughout the study and were not separated or reorganized according to study condition. Experimental classes received the active gamification program, whereas control classes continued with the standard Physical Education curriculum. Teachers were instructed not to share intervention-related content across study conditions to minimize contamination. Teachers delivering the intervention received two weeks of training in the gamification methodology before the program began, whereas control teachers followed the standard curriculum. Throughout the intervention, researchers monitored implementation fidelity through direct observations and weekly checklists to verify adherence to the protocol, session timing, student participation, and attendance.

Each of the six participating classes attended three 45 min Physical Education sessions per week during its regularly scheduled Physical Education periods within the school timetable. The classes remained intact throughout the study, and the experimental or control condition was delivered to each class during its corresponding Physical Education lessons.

### 2.6 Sample size calculation

An *a priori* sample size calculation was performed using G * Power (version 3.1.9.7; Heinrich Heine University Düsseldorf, Düsseldorf, Germany). The calculation was based on an *F* test for the group × time interaction in a repeated-measures design with two groups and two measurement occasions. Assuming a small-to-moderate effect size (*f* = 0.134), a significance level of *α* = 0.05, a statistical power of 80% (1 − *β* = 0.80), a correlation of 0.50 between repeated measurements, and a nonsphericity correction of *ε* = 1.0, the estimated minimum sample size was approximately 112 participants (56 per group). To account for an anticipated attrition rate of approximately 7%, the recruitment target was increased to 120 participants. A total of 120 children were ultimately recruited, with 60 participants allocated to each group.

### 2.7 Data analysis

Descriptive statistics were calculated for all study variables and are presented as means and standard deviations for continuous variables and frequencies and percentages for categorical variables. Given the hierarchical and longitudinal structure of the data, linear mixed-effects models (LMMs) were used to evaluate intervention effects while accounting for the non-independence of observations. Separate LMMs were fitted for concentration, selective attention, immediate visual memory, and creativity. Time (pre- vs. post-intervention), group (EG vs. CG), and the group × time interaction were included as fixed effects, with the group × time interaction representing the primary test of the intervention effect. Classroom was included as a random intercept to account for the clustering of students within intact classes. Repeated pre- and post-intervention measurements were modeled within participants using a compound-symmetry covariance structure. Models were estimated using restricted maximum likelihood (REML). Estimated marginal means and 95% confidence intervals were obtained from the fitted models to characterize changes over time within each study group and the differential change between groups. Planned pairwise *post hoc* comparisons were performed to examine between-group differences at each time point (EG vs. CG at pre- and post-intervention) and within-group changes over time (pre- vs. post-intervention separately for the EG and CG). To account for multiple comparisons, *p*-values for these pairwise comparisons were adjusted using the Bonferroni correction. Given the small number of classroom clusters (six classes; three per study condition), classroom-level random-effect estimates were interpreted cautiously, and a parsimonious random-effects structure was used. Effect sizes were calculated using Cohen's *d*, with values of approximately 0.20, 0.50, and 0.80 interpreted as small, moderate, and large effects, respectively (30). Statistical significance was set at *p* < .05. All analyses were performed using IBM SPSS Statistics, version 22.0 (IBM Corp., Armonk, NY, USA).

## 3 Results

The participants had a mean age of 9.40 ± 0.98 years, with 44.2% being boy and 55.8% being girls. No significant baseline differences were found between the CG and EG in concentration, selective attention, memory, or creativity (all *p* > .05) (Table 1). Students attended a minimum of 98.3% of scheduled intervention sessions, with no incidents related to injuries or adverse reactions recorded throughout the intervention period.

**Table 1:** Preintervention sociodemographic characteristics of the participants.

Characteristics of the participants		Total (*n* = 120)	Experimental (*n* = 60)	Control (*n* = 60)	*p*-value
Age		9.40 ± 0.98	9.25 ± 0.93	9.55 ± 1.02	*0*.*094*
Sex	Boy	53 (44.20)	26 (49.10)	27 (50.90)	*0*.*856*
	Girl	67 (55.80)	34 (50.70)	33 (49.30)	
	No studies	6 (5.00)	4 (66.70)	2 (33.30)	
Mother's studies (%)	Primary studies	17 (14.20)	7 (41.20)	10 (58.80)	*0*.*526*
	Secondary studies	46 (38.30)	20 (43.50)	26 (56.50)	
	University studies	51 (42.50)	29 (56.90)	22 (43.10)	
Concentration_D2		59.26 ± 9.11	58.43 ± 9.11	60.08 ± 9.12	*0*.*323*
Selective attention_D2		66.74 ± 2.69	67.10 ± 3.31	66.38 ± 1.83	*0*.*145*
Memory_RIAS		9.42 ± 3.25	9.37 ± 3.38	9.47 ± 3.15	*0*.*867*
Creativity_CREA		44.84 ± 9.17	44.22 ± 7.83	45.47 ± 10.37	*0*.*458*

Quantitative variables are presented as mean and standard deviation. Qualitative variables are presented as frequency and percentage.

### 3.1 Concentration

The linear mixed-effects model showed a significant group × time interaction for concentration, *F*(1, 236) = 4.60, *p* = 0.033. A significant main effect of time was also observed, *F*(1, 236) = 5.22, *p* = 0.023, whereas the main effect of group was not significant, *F*(1, 236) = 0.57, *p* = 0.453. Estimated marginal means indicated that concentration remained essentially stable in the control group (60.08 at pre-intervention and 60.25 at post-intervention), whereas it increased in the experimental group from 58.43 to 63.68. The estimated differential change between groups was 5.08 points (95% CI: 0.41 to 9.76). Bonferroni-adjusted pairwise comparisons showed no significant between-group difference at pre-intervention (mean difference = 1.65, 95% CI: −1.65 to 4.95, *p* = 0.326), whereas at post-intervention the experimental group scored significantly higher than the control group (mean difference = 3.43, 95% CI: 0.13 to 6.74, *p* = 0.042, Cohen’s *d* = 0.37). Within-group comparisons showed no significant pre-to-post change in the control group (mean change = 0.17, 95% CI: −3.14 to 3.47, *p* = 0.921, Cohen’s d = 0.02), whereas concentration significantly increased in the experimental group (mean change = 5.25, 95% CI: 1.95 to 8.55, *p* = 0.002, Cohen’s d = 0.57) (Figure 2).

**Figure 2:**
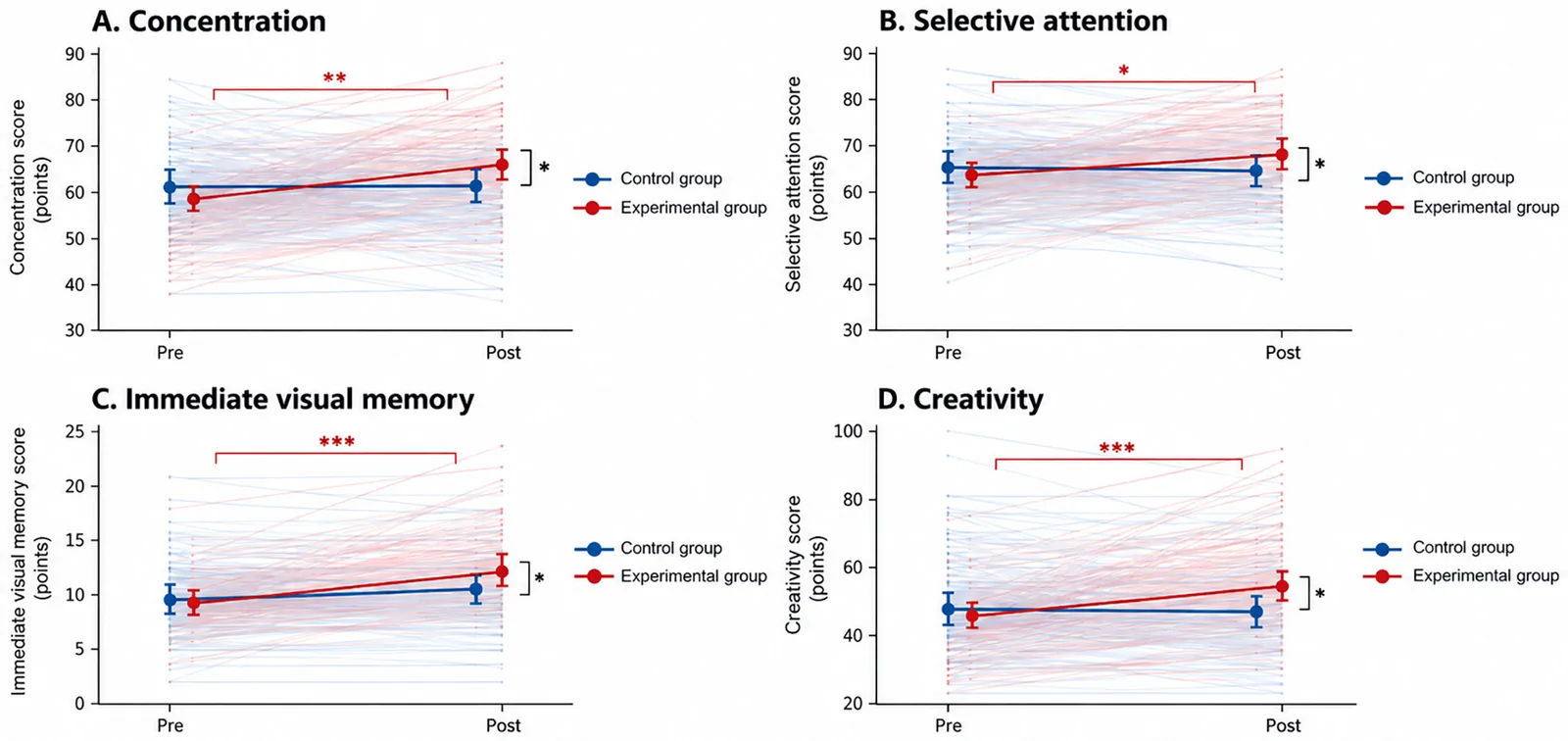
Individual trajectories and model-estimated changes in cognitive outcomes following the intervention. Thin lines represent individual participant trajectories from pre- to post-intervention. Points and error bars represent estimated marginal means (EMMs) and their corresponding 95% confidence intervals derived from the linear mixed-effects models for the control and experimental groups. **(A)** Concentration; **(B)** selective attention; **(C)** immediate visual memory; **(D)** creativity.

### 3.2 Selective attention

The linear mixed-effects model showed a significant group × time interaction for selective attention, *F*(1, 236) = 9.26, *p* = 0.003. Estimated marginal means indicated that selective attention decreased slightly in the control group (66.38 at pre-intervention and 65.42 at post-intervention), whereas it increased in the experimental group from 67.10 to 68.40. The estimated differential change between groups was 2.27 points (95% CI: 0.80 to 3.73). Bonferroni-adjusted pairwise comparisons showed no significant between-group difference at pre-intervention (mean difference = 0.72, 95% CI: −0.32 to 1.75, *p* = 0.175), whereas at post-intervention the experimental group scored significantly higher than the control group (mean difference = 2.98, 95% CI: 1.95 to 4.02, *p* < 0.001, Cohen’s *d* = 1.03). Within-group comparisons showed no significant pre-to-post change in the control group (mean change = −0.97, 95% CI: −2.00 to 0.07, *p* = 0.068, Cohen’s *d* = 0.34), whereas selective attention significantly increased in the experimental group (mean change = 1.30, 95% CI: 0.26 to 2.34, *p* = 0.014, Cohens’s *d* = 0.45) (Figure 2).

### 3.3 Memory

The linear mixed-effects model showed a significant group × time interaction for memory, *F*(1, 236) = 4.37, *p* = 0.038. Estimated marginal means indicated that memory scores increased from 9.47 to 10.20 in the control group and from 9.37 to 11.62 in the experimental group. The estimated differential change between groups was 1.52 points (95% CI: 0.09 to 2.95). Bonferroni-adjusted pairwise comparisons showed no significant between-group difference at pre-intervention (mean difference = 0.10, 95% CI: −0.91 to 1.11, *p* = 0.846), whereas at post-intervention the experimental group scored significantly higher than the control group (mean difference = 1.42, 95% CI: 0.41 to 2.43, *p* = 0.006, Cohen’s *d* = 0.51). Within-group comparisons showed no significant pre-to-post change in the control group (mean change = 0.73, 95% CI: −0.28 to 1.74, *p* = 0.154, Cohen’s = 0.26), whereas memory significantly increased in the experimental group (mean change = 2.25, 95% CI: 1.24 to 3.26, *p* < 0.001, Cohen’s *d* = 0.80) (Figure 2).

### 3.4 Creativity

The linear mixed-effects model showed a significant group × time interaction for creativity, *F*(1, 232.044) = 18.51, *p* < 0.001. Estimated marginal means indicated that creativity remained relatively stable in the control group (45.48 at pre-intervention and 44.70 at post-intervention), whereas it increased in the experimental group from 44.22 to 53.00. The estimated differential change between groups was 9.57 points (95% CI: 5.19 to 13.95). Bonferroni-adjusted pairwise comparisons showed no significant between-group difference at pre-intervention (mean difference = 1.27, 95% CI: −3.16 to 5.69, *p* = 0.533), whereas at post-intervention the experimental group scored significantly higher than the control group (mean difference = 8.30, 95% CI: 3.88 to 12.73, *p* = 0.002, Cohen’s *d* = 0.96). Within-group comparisons showed no significant pre-to-post change in the control group (mean change = −0.78, 95% CI: −3.88 to 2.32, *p* = 0.619, Cohen’s *d* = 0.009), whereas creativity significantly increased in the experimental group (mean change = 8.78, 95% CI: 5.69 to 11.88, *p* < 0.001, Cohen’s *d* = 1.02) (Figure 2).

### 3.5 Random-effects estimates

Random-effects parameter estimates indicated minimal between-classroom variability across the cognitive outcomes (Table 2). The estimated classroom random-intercept variance was 0.000 (SD = 0.000) for concentration, selective attention, and memory, with residual variances of 84.354, 8.324, and 7.893, respectively, resulting in ICCs of 0.000 for these outcomes. For creativity, the classroom random-intercept variance was 1.996 (SD = 1.413), with a residual variance of 74.182, resulting in an ICC of 0.026. Thus, approximately 2.6% of the total variance in creativity was attributable to between-classroom differences, whereas the estimated between-classroom variance was negligible for the remaining outcomes.

**Table 2:** Random-effects parameter estimates and intraclass correlation coefficients for the cognitive outcomes.

Outcome	Classroom variance	Classroom SD	Residual variance	ICC
Concentration	0.000	0.000	84.354	0.000
Selective attention	0.000	0.000	8.324	0.000
Memory	0.000	0.000	7.893	0.000
Creativity	1.996	1.413	74.182	0.026

SD, standard deviation; ICC, intraclass correlation coefficient. ICC was calculated as classroom variance/(classroom variance + residual variance). A classroom variance estimate of 0.000 indicates that the estimated between-classroom variance was at the boundary of zero.

## 4 Discussion

This study evaluated the effects of a 12-week physically active gamification program on concentration, selective attention, creativity, and immediate visual memory in primary school children. Overall, the EG showed more favorable pre–post trajectories than the CG across the four cognitive outcomes, although the magnitude and pattern of change differed between measures. Concentration improved in the EG, whereas the CG remained relatively stable. Selective attention increased in the EG but showed a small decrease in the CG. Immediate visual memory improved in both groups, although the increase was greater in the EG. Finally, creativity showed a marked improvement in the EG, whereas the CG remained comparatively stable. This heterogeneous pattern suggests that the intervention did not affect all cognitive outcomes to the same extent and that each outcome should be interpreted according to the specific cognitive process assessed. For descriptive purposes, the pre- and post-intervention values presented below as mean ± SD represent raw observed descriptive statistics rather than model-adjusted estimates. Inferential conclusions regarding intervention effects are based on the linear mixed-effects models and their corresponding estimated marginal means and pairwise contrasts reported in the Results section.

Concentration and selective attention are essential cognitive skills for learning, enabling students to sustain focus, prioritize relevant information, and process tasks efficiently (31–33). In the present study, concentration increased from 58.43 ± 9.11 to 63.68 ± 9.44 in the EG, whereas scores remained comparatively stable in the CG (60.08 ± 9.12 to 60.25 ± 9.07). Selective attention followed a somewhat different trajectory, increasing from 67.10 ± 3.31 to 68.40 ± 3.93 in the EG while decreasing slightly from 66.38 ± 1.83 to 65.42 ± 1.88 in the CG. Thus, although both attentional outcomes favored the experimental condition, the pattern of change was not identical: the concentration effect was primarily characterized by improvement in the EG, whereas the selective-attention effect reflected both improvement in the EG and a small decline in the CG.

Previous studies have suggested that gamified learning environments may support attentional involvement through characteristics such as clear goals, feedback, challenges, and rewards (34, 35). Likewise, physically active tasks involving directed attention have been associated with cognitive processes related to self-regulation and cognitive control (36, 37). These findings provide a possible theoretical framework for interpreting the present results. However, motivation, engagement, mental fatigue, and neural activation were not directly assessed in the present study. Consequently, the observed improvements in concentration and selective attention cannot be attributed to changes in these mechanisms on the basis of the current data.

The small reduction in selective attention observed in the CG should also be interpreted cautiously. Because no measure of fatigue, motivation, classroom conditions, or other contextual factors was collected, the present study cannot determine why this decrease occurred. It may reflect normal variability across repeated assessments or other unmeasured influences rather than a meaningful deterioration in attention. Importantly, the intervention effect should therefore be interpreted from the overall differential trajectory between groups rather than by assuming that active gamification prevented a specific mechanism responsible for the decline in the CG.

These findings can also be considered within the distinction between general and domain-specific cognitive processes. The outcomes assessed in this study reflect general cognitive functions rather than subject-specific academic performance. Although theoretical frameworks such as the dual-process perspective may help contextualize changes in general cognitive functioning (38), no domain-specific academic outcomes were assessed. It therefore remains unknown whether the improvements observed in concentration and selective attention translate into better performance in mathematics, language, or other academic subjects. This distinction is relevant because classroom-based physically active interventions may produce different effects depending on whether their cognitive demands are directly aligned with specific academic content (38).

Immediate visual memory showed another distinct pattern. Scores increased from 9.37 ± 3.38 to 11.62 ± 2.64 in the EG, while the CG also improved, from 9.47 ± 2.99 to 10.20 ± 1.81. Thus, unlike concentration and selective attention, improvement was not restricted to the experimental condition, although the magnitude of change was greater in the EG. This finding is consistent with previous research reporting favorable associations between physically active, cognitively engaging activities and memory-related performance in children and adolescents (39). Nevertheless, the improvement observed in both groups indicates that the result should not be interpreted as an effect occurring exclusively in children exposed to active gamification.

The characteristics of the memory assessment also require careful consideration when interpreting this finding. The task required children to recall visual information immediately after a brief presentation and therefore specifically assessed immediate visual recall, rather than long-term memory, memory consolidation, or broader memory functioning. Moreover, different sets of cards were administered at pre- and post-test to minimize recall and practice effects associated with repeated exposure to the same stimuli. Although previous research has proposed neural and cognitive mechanisms through which physical and cognitively demanding activities may influence memory processes (14, 40), these mechanisms were not assessed in the present study. Accordingly, our findings support an improvement in immediate visual recall performance but do not provide evidence regarding long-term retention, memory consolidation, or underlying neural changes.

Creativity showed the most pronounced differential trajectory. Baseline scores were similar between the CG and EG (45.47 ± 10.37 vs. 44.22 ± 7.83, respectively). Following the intervention, creativity increased substantially in the EG to 53.00 ± 6.94, whereas scores in the CG remained comparatively stable (44.68 ± 9.23 at post-test). The significant group × time interaction therefore indicates a markedly more favorable trajectory in the EG. Previous research has reported mixed findings regarding the effects of gamification on creativity. Some studies suggest that gamified learning environments can promote divergent thinking by encouraging exploration and flexible problem-solving (41), whereas others indicate that effects may depend on intervention design, particularly when competitive or strongly goal-oriented structures are used (42). Collaboration and problem-solving within gamified environments have also been proposed as potentially relevant features for creative performance (43).

In the present intervention, problem-solving, route planning, riddles, strategic tasks, and collaboration were not additional cognitive-training components delivered alongside gamification; rather, they constituted the challenges and missions through which the gamified methodology was implemented. Repeated engagement with these types of activities provides a plausible context for the improvement observed in CREA performance. However, because these elements formed part of an integrated intervention and were not manipulated or assessed independently, the present study cannot determine which specific gamification features contributed to the observed creativity effect.

The different trajectories observed across the four outcomes are noteworthy. The intervention produced comparatively pronounced between-group differences in creativity and selective attention, whereas the patterns for concentration and immediate visual memory were more modest. Several explanations are possible, including differences in the cognitive demands elicited by the intervention, the sensitivity of the assessment instruments to change, or the extent to which each cognitive process was engaged during the gamified activities. However, these possibilities remain theoretical because the study did not directly assess the cognitive demands of individual activities or potential mediators such as motivation, engagement, perceived cognitive load, or mental fatigue. Consequently, the present results should not be interpreted as evidence that active gamification acts through any specific psychological or neural mechanism. Future studies incorporating these measures could help explain why some cognitive outcomes appear more responsive to physically active gamification than others.

Several limitations should be considered when interpreting these findings. First, all participants were recruited from a single public primary school in eastern Andalusia, Spain, with a predominantly middle socioeconomic background. This single-school setting limits the external validity and generalizability of the findings, as the results may partly reflect characteristics specific to this educational context. Replication across multiple schools, socioeconomic contexts, and geographical regions is therefore warranted. Second, although the analyses accounted for the hierarchical structure of the data by including classroom as a random effect, only six classroom clusters (three per study condition) were available. The small number of clusters limits the precision with which between-class variability can be estimated and warrants caution when interpreting classroom-level random effects. Future cluster-randomized studies should include a larger number of schools and classroom clusters. Third, participants' perceptions of the intervention were not assessed. Measures of enjoyment, motivation, perceived challenge, cognitive load, or stress could provide valuable information about how children experienced the program and whether these factors are associated with cognitive outcomes. Fourth, because the intervention was designed as an integrated physically active gamification program, the study evaluates its overall effect and does not allow the contribution of individual gamification elements or specific types of challenges to be isolated. Finally, the cognitive measures used represent specific aspects of cognitive functioning. In particular, the memory task assessed immediate visual recall and should not be generalized to broader memory processes or long-term retention.

Despite these limitations, the study used a cluster-randomized controlled design and implemented the intervention within regular school physical education, providing ecologically relevant evidence regarding the potential cognitive effects of physically active gamification. The findings indicate that a 12-week physically active gamification program produced more favorable changes in concentration, selective attention, immediate visual memory, and creativity compared with standard physical education in this sample of primary school children. Rather than suggesting a uniform enhancement of cognition, the results indicate that the magnitude and pattern of change differed across cognitive outcomes. Further multicenter cluster-randomized studies should examine the reproducibility of these findings and directly assess the psychological, cognitive, and contextual mechanisms that may account for the observed effects. Finally, cognitive outcomes were assessed immediately after completion of the 12-week intervention, and no follow-up assessments were conducted. Therefore, the present study cannot determine whether the observed improvements in concentration, selective attention, immediate visual memory, and creativity were maintained after the intervention ended. Future studies should incorporate longitudinal follow-up assessments (e.g., several weeks or months after intervention completion) to examine the retention and sustainability of cognitive changes over time. In addition, the cognitive measures used represent specific aspects of cognitive functioning. In particular, the memory task assessed immediate visual recall and should not be generalized to broader memory processes or long-term retention.

## 5 Conclusion

This study provides evidence that a 12-week physically active gamification program implemented during regular physical education lessons can positively influence cognitive functioning in primary school children. Compared with standard physical education, the intervention produced more favorable changes in concentration, selective attention, immediate visual memory, and creativity, although the magnitude and pattern of these changes differed across cognitive outcomes. These findings suggest that physically active gamification may represent a useful educational approach for promoting specific cognitive processes within the school setting. Importantly, the results should be interpreted in relation to the specific outcomes assessed and should not be generalized to broader cognitive or academic functioning. In particular, the memory outcome reflected immediate visual recall rather than long-term memory, and no academic performance measures or potential psychological and neural mediators were assessed. Furthermore, because the intervention integrated multiple gamification elements within physically active tasks, the contribution of individual components cannot be isolated. Future research should replicate these findings in larger and more diverse samples and examine the mechanisms underlying the differential responses observed across cognitive outcomes.

## Data Availability

The raw data supporting the conclusions of this article will be made available by the authors, without undue reservation.
