# Supplementary material for: Effects of a physically active gamification program on cognitive function in primary school children
Source: Front Sports Act Living. 2026 Oct 8;8:1967341. doi: 10.3389/fspor.2026.1967341 (PMC13687043; doi:10.3389/fspor.2026.1967341)
Supplement: Supplementary file 1 [file Datasheet1.pdf]

Supplementary Table S1. Detailed description of the 12-week physically active gamification intervention

Phase	Week	Session	Physical tasks and motor objectives	Cognitive processes engaged	Gamification dynamics and mechanics
1. Explorers in Action	1. Introduction to Exploration	1. Discovering the Territory	Teams completed a physical task (e.g., hopping on one foot, spinning, or touching their toes) before running to a designated area and claiming it by placing a marker or flag. Objectives: spatial awareness and collaboration.	Spatial awareness; attention; decision-making; collaboration	Explorer narrative; territory-conquest mission; points for successfully claimed areas; team competition; immediate feedback
		2. Explorers on the Road	Teams used hoops or blocks as "bridges" to cross "rivers" without touching the ground, moving the materials cooperatively. Objectives: coordination and planning.	Planning; problem-solving; spatial processing; cooperation	Explorer narrative; cooperative mission; rule-based challenge; progressive difficulty; XP; immediate feedback
		3. Explorers of Movement	Students followed footprint-marked paths while performing movements such as jumping or crouch walking. Objectives: agility and endurance.	Attention; movement sequencing; motor planning	Explorer narrative; progressive movement challenge; XP; symbolic recognition; immediate feedback
	2. Coordination Challenges	4. Tower Challenge	Teams transported materials (e.g., hoops and cones) while maintaining balance or negotiating obstacles and used them to construct a tower. Objectives: balance and coordination/teamwork.	Planning; attention; cooperation	Team challenge; points linked to tower height and error-free transport; collective ranking; immediate feedback
		5. Team Explorers	Teams negotiated obstacles to collect "gems" and return them to the starting point. Objectives: agility and cooperation.	Observation; spatial orientation; planning; cooperation	Treasure-collection mission; XP; team progression; symbolic rewards; immediate feedback
		6. The Explorer's Race	Relay-style circuit involving jumping, zigzag running, and object transport. Objectives: speed and endurance.	Attention; sequencing; teamwork	Explorer race; team challenge; XP; collective ranking; progressive difficulty; immediate feedback

		7. The Explorer's Labyrinth	Students navigated an arrow-marked maze/circuit while avoiding restricted zones and completing the route as efficiently as possible. Objectives: agility, planning and teamwork.	Planning; spatial orientation; problem-solving; attention	Maze/exploration mission; rule-based challenge; time/performance goal; XP; immediate feedback
	3. Overcoming Explorer Challenges	8. The Lost Explorer	Students followed a marked route while avoiding restricted areas, with classmates providing guidance. Objectives: coordination, planning and teamwork.	Planning; observation; spatial processing; communication	Lost-explorer narrative; cooperative roles; guidance-based mission; XP; symbolic recognition; immediate feedback
		9. Hunting for Lost Objects	Teams followed clues to locate hidden objects while completing physical actions such as running, jumping, and crouching. Objectives: agility, endurance and observation.	Observation; attention; problem-solving; spatial search	Treasure-hunt mission; clue-based progression; XP; team ranking; symbolic rewards; immediate feedback
		10. Treasure Race	Multi-station circuit combining jumping, balance-based object transport and zigzag running to reach the "treasure." Objectives: balance, agility, coordination and speed.	Attention; sequencing; planning; cooperation	Treasure narrative; station-based challenge; XP; collective ranking; progressive difficulty; immediate feedback
	4. Explorer Consolidation	11. Explorer Challenge	Combined target-throwing and balance activities with puzzle-solving tasks. Objectives: agility, coordination and integrated motor performance.	Problem-solving; strategy; attention	Explorer challenge; puzzle mechanics; progressive challenge; XP; symbolic recognition; immediate feedback
		12. The Treasure Party	Clue-based physical challenges including jumping, short races and riddles, integrating skills practiced during the phase. Objectives: consolidation of motor skills and teamwork.	Problem-solving; attention; flexible thinking; cooperation	Final explorer mission; clues and riddles; accumulated XP; team ranking; symbolic rewards; immediate feedback
Phase 2. Movement Heroes	5. Hero Training	13. The Hero's Journey	Dynamic obstacle circuit requiring continuous movement and adaptation. Objectives: endurance and coordination.	Attention; sequencing; motor planning	Hero narrative and role; mission-based challenge; XP; progressive

				difficulty; symbolic recognition; immediate feedback
	14. The Sleeping Dragon Challenge	Teams transported objects quietly and cooperatively toward a goal while avoiding actions that would “wake” the dragon. Objectives: teamwork, coordination and motor control.	Attention; planning; cooperation	Sleeping-dragon narrative; rule-based cooperative challenge; XP; team progression; immediate feedback
	15. Defending the Fortress	Students threw balls from a designated distance to knock down towers. Objectives: throwing accuracy and coordination/cooperation.	Attention; strategy; visuomotor planning	Fortress-defense narrative; points for successful targets; team ranking; symbolic rewards; immediate feedback
6. Kingdom	16. Dragon Race	Students completed a circuit while collecting and transporting “gems.” Objectives: speed, coordination and endurance.	Attention; spatial orientation; planning	Dragon/hero narrative; resource collection; XP; team ranking; progressive challenge; immediate feedback
	17. Rescue of the Kingdom	Teams cooperatively transported a large object through an obstacle course using available/simple tools. Objectives: teamwork and coordination.	Planning; problem-solving; cooperation	Kingdom-rescue mission; cooperative challenge; XP; symbolic rewards; immediate feedback
	18. Defense of the Kingdom	One team guarded a treasure while the other attempted to retrieve it without being caught. Objectives: agility, strategy and teamwork.	Strategy; attention; decision-making; cooperation	Kingdom-defense narrative; opposing team roles; treasure objective; XP; collective ranking; immediate feedback
7. Advanced Collaboration Challenges	19. The Resource Mission	Teams navigated an obstacle circuit to retrieve resources distributed throughout the playing area. Objectives: coordination, teamwork and collaboration.	Planning; attention; spatial orientation; cooperation	Rescue/resource mission; collection mechanic; XP; team progression; symbolic recognition; immediate feedback

		20. Protecting the Fortress	Teams defended their base while completing a continuous physical circuit. Objectives: endurance, collaboration and teamwork.	Attention; strategy; cooperation	Fortress-defense narrative; team roles; XP; collective ranking; progressive challenge; immediate feedback
		21. The Test of Heroes	Relay-based circuit with several stations emphasizing speed and accuracy. Objectives: agility, speed and endurance.	Attention; sequencing; teamwork	Heroic test; points/XP linked to speed and accuracy; team ranking; symbolic rewards; immediate feedback
	8. The Final Challenge of the Heroes	22. The Last Battle for the Kingdom	Teams transported "magic resources" to their base while negotiating physical obstacles. Objectives: endurance, coordination and teamwork.	Planning; strategy; cooperation	Final-battle narrative; resource-collection mission; XP; collective ranking; progressive difficulty; immediate feedback
		23. The Race Against the Dragon	Timed obstacle race requiring students to complete the course efficiently. Objectives: speed, endurance and planning.	Attention; planning; decision-making	Dragon-race narrative; timed challenge; XP; team progression/ranking; immediate feedback
		24. The Triumph of the Heroes	Comprehensive multi-station circuit integrating throwing, agility and speed tasks practiced during the phase. Objectives: agility, cooperation and integration of motor skills.	Attention; planning; strategy; cooperation	Final hero challenge; accumulated XP; collective ranking; symbolic achievement recognition; immediate feedback
Phase 3. Guardians of Health	9. Guardian Challenges	25. Protectors of the Fortress	Teams attempted to knock down opposing towers using balls. Objectives: strength, throwing accuracy, coordination and teamwork.	Attention; strategy; visuomotor planning	Guardian/fortress narrative; points for successful hits; team ranking; symbolic rewards; immediate feedback
		26. The Balance Test	Teams performed synchronized squats while maintaining tension on a shared rope. Objectives: balance, strength, coordination and cooperation.	Attention; synchronization; cooperation	Guardian challenge; cooperative performance goal; XP; symbolic recognition; immediate feedback

	27. The Guardians' Race	Relay-style obstacle course completed sequentially by team members. Objectives: endurance, agility and teamwork.	Attention; sequencing; teamwork	Guardian race; relay mechanics; XP; collective ranking; progressive difficulty; immediate feedback
10. Guardians' Missions	28. The Great Recovery	Teams moved around a circuit to collect as many "lost resources" as possible within a time limit. Objectives: speed and endurance.	Attention; spatial search; decision-making	Recovery/resource narrative; collection mechanic; time-limited challenge; XP; team ranking; immediate feedback
	29. The Kingdom Bridges	Teams transported blocks across an area using ropes or hoops without touching the ground. Objectives: coordination and cooperation.	Problem-solving; planning; creativity; cooperation	Strategic-transfer mission; rule-based cooperative challenge; XP; progressive difficulty; immediate feedback
	30. The Protective Shield	Teams moved objects cooperatively to construct a collective protective "shield" or barrier. Objectives: strength, teamwork and coordination.	Planning; problem-solving; cooperation	Construction mission; collective achievement; XP; symbolic recognition; immediate feedback
11. Preparation for the Final Battle	31. The Guardians' Endurance	Team relay in which participants alternated between completing a physical circuit and recovery periods. Objectives: endurance, agility and teamwork.	Attention; sequencing; pacing; cooperation	Guardian endurance mission; relay mechanics; XP; team ranking; progressive challenge; immediate feedback
	32. The Kingdom Strategy	Teams designed and constructed a fortress using blocks, ropes and hoops in preparation for the final activities. Objectives: coordination and teamwork.	Planning; creativity; problem-solving; collaborative decision-making	Strategic construction mission; team planning; XP; symbolic recognition; immediate feedback
	33. Final Defense Rehearsal	Final Battle Simulation combining throwing, balance and relay tasks. Objectives: speed, accuracy, coordination and integrated motor performance.	Strategy; attention; planning; cooperation	Final-battle simulation; progressive challenge; XP; collective ranking; symbolic rewards; immediate feedback

	34. The Assault on the Towers	Multi-station final challenge involving tower throwing, synchronized squats with a rope and obstacle relays. Objectives: strength, speed, agility and teamwork.	Strategy; attention; sequencing; cooperation	Final-battle narrative; multi-stage challenge; points/XP; team ranking; immediate feedback
12. The Final Battle	35. The Unbreakable Fortress	Teams used available materials to construct a fortress collaboratively. Objectives: coordination, teamwork and integrated motor performance.	Problem-solving; creativity; planning; cooperation	Final construction mission; collective achievement; XP; symbolic recognition; immediate feedback
	36. Crowning of the Guardians	Final activities and celebration recognizing the teams' accumulated achievements throughout the intervention. Objectives: teamwork and consolidation of the program experience.	Cooperation; reflection on collective achievement	Narrative resolution; accumulated XP; final team ranking; symbolic rewards and recognition; crowning as "Guardians of Health"
